# The effects of family resilience and body image on psychosocial adaptation among young and middle-aged breast cancer patients

**DOI:** 10.3389/fpsyg.2026.1909339

**Published:** 2026-09-23

**Authors:** Yanhong Zhou, Wenhui Geng, Fengxia Liu, Xiaomeng Lu, Xiaoling Tian

**Affiliations:** 1 Department of Radiation Oncology, The Fourth Hospital of Hebei Medical University, Shijiazhuang, Hebei, China; 2 Department of Nursing, The Fourth Hospital of Hebei Medical University, Shijiazhuang, Hebei, China

**Keywords:** body image, breast cancer, family resilience, psychosocial adaptation, structural equation model, young and middle-aged

## Abstract

**Aim:**

To determine how family resilience and body image are related to psychosocial adaptation in young and middle-aged patients with breast cancer and whether body image functions as a mediator.

**Background:**

Social support is known to be relevant to adaptation after breast cancer. Less is known about the pathway linking family resilience to psychosocial adaptation through body image.

**Methods:**

Questionnaire data were obtained from 229 postoperative patients selected by convenience sampling. Family resilience, body image, and psychosocial adaptation were measured using the Chinese versions of the Family Resilience Scale, Body Image Scale, and Psychosocial Adaptation Scale for Breast Cancer, respectively. The hypothesized associations and indirect pathway were evaluated by structural equation modeling.

**Results:**

Valid data were available for 220 of the 229 participants (96.07%). Family resilience, body image, and psychosocial adaptation had mean scores of 102.34 ± 12.93, 7.19 ± 5.91, and 181.10 ± 27.97, respectively. Family resilience was negatively correlated with body image (*r* = −0.292, *P* < 0.01) and positively correlated with psychosocial adaptation (r = 0.480, P < 0.01). Of the total association between family resilience and psychosocial adaptation, 67.58% was attributable to the direct pathway and 32.42% to the indirect pathway through body image.

**Conclusion:**

Patients reporting stronger family resilience also showed better psychosocial adaptation. Part of this association operated through body image. Interventions addressing both family resilience and body image may be useful for young and middle-aged patients with breast cancer.

**Relevance to clinical practice:**

Assessment of psychosocial adaptation should consider both the patient's family environment and concerns about physical appearance. Family-based support may assist patients in managing body image distress and adapting to life after breast cancer treatment.

## 1 Introduction

Breast cancer accounted for approximately 2.309 million newly diagnosed cancer cases and 666,000 deaths worldwide in 2022. Data from the International Agency for Research on Cancer (IARC) showed that breast cancer ranked second in cancer incidence and fourth in cancer-related mortality in 2022 (Bray et al., 2024). Of the global total, China accounted for 21.11% of new cases and 11.96% of deaths. Women aged 18–59 years comprise approximately 53.9% of Chinese patients with breast cancer (Han et al., 2024). For these women, completing treatment does not mean that all difficulties have ended. Changes in appearance, hair loss, sexual dysfunction, limitations in work and household activities, and treatment-related financial pressure may continue after surgery (Singleton et al., 2022; Álvarez-Pardo et al., 2022; Baudry et al., 2022; Geng et al., 2025).

In this study, psychosocial adaptation refers to the way patients respond to the emotional and social changes associated with breast cancer (Zhang et al., 2023). Patients with good psychosocial adaptation are better able to cope with illness-related physical and psychological changes, maintain self-efficacy and social identity, experience less anxiety, depression, and social withdrawal, and participate more actively in family and social activities (Mahin et al., 2025). However, a previous trajectory study found persistent severe maladaptation in 22.5% of young and middle-aged patients and persistent moderate maladaptation in 50.4% (Zhang et al., 2024). These findings indicate that difficulties with adaptation remain common and that their associated factors warrant attention.

Family resilience refers to a family's ability to withstand adversity, recover from it, and grow through the experience (Walsh, 1996). Its main components include family belief systems, communication processes, and organizational processes (Cui et al., 2024). In the context of breast cancer, these resources can affect how family members respond to treatment demands and support the patient. Earlier studies reported relationships between family resilience and patients' physical and emotional symptoms (Cui et al., 2025; Li et al., 2023; Chen et al., 2024).

Patients' responses to changes in appearance are captured by the concept of body image (Grogan, 2021). Such changes may influence how a woman views herself and interacts with others. Distress concerning body image has been related to emotional health, social functioning, prognosis, and quality of life (An et al., 2022; Pereira et al., 2021). Nevertheless, family resilience and body image have usually been examined separately.

Bronfenbrenner's ecological systems theory offers a basis for considering them together. The theory describes the microsystem, mesosystem, exosystem, and macrosystem as connected levels of the individual's environment. Of these, the microsystem is the setting in which direct interactions occur. The family belongs to this level and is especially relevant during recovery from breast cancer. How family members communicate, solve problems, and respond emotionally may influence both the patient's view of her body and her adaptation to illness. This interpretation is supported by reported relationships of social support and family functioning with psychosocial adaptation (Tao et al., 2023; Min et al., 2025)_._

Body image may be involved in the relationship between the family environment and psychosocial adaptation (Farnicka et al., 2026). This possibility is relevant to young and middle-aged women, who may need to balance treatment and recovery with employment, childcare, intimate relationships, and concerns about appearance. Negative feelings about bodily changes can be accompanied by lower self-esteem, anxiety, depression, and avoidance of social contact. Existing studies have provided information about the separate relationships among these variables, but less attention has been paid to their relationship within one model. We therefore examined whether body image was statistically involved in the association between family resilience and psychosocial adaptation. Based on this theoretical framework and logical deduction, we propose the following hypotheses:

H1: Family resilience is positively associated with psychosocial adaptation in breast cancer patients.

H2: Family resilience is negatively associated with body image distress.

H3: Body image distress is negatively associated with psychosocial adaptation.

H4: The relationship between family resilience and psychosocial adaptation is mediated by body image.

## 2 Materials and methods

### 2.1 Ethical approval

Ethical review was granted by the Medical Ethics Committee of the Fourth Hospital of Hebei Medical University under approval number 2025KS136. All research procedures complied with the Declaration of Helsinki. Before entering the study, every participant signed an informed consent form. Measures were taken to preserve participant privacy during all stages of the research.

### 2.2 Study design and participants

Patients attending the Breast Radiotherapy Clinic of the Fourth Hospital of Hebei Medical University between September 2025 and February 2026 formed the sampling population for this cross-sectional study. A non-probability convenience approach was used for recruitment. To be eligible, an individual had to (1) be at least 18 but younger than 60 years; (2) have a pathological diagnosis of breast cancer (BC); (3) have completed surgery or chemotherapy; (4) be able to read and comprehend the questionnaire; and (5) provide written informed consent. Individuals were not eligible if they had another malignant tumor, a serious organic disease, a psychiatric or cognitive disorder, or were undergoing psychological treatment.

Sample size was determined according to Kendall's recommendation of recruiting 5–10 participants for each observed variable (Jobst et al., 2023). On the basis of the 24 items used in the calculation and an anticipated invalid-response rate of 10%, the planned enrollment range was 166–267. Enrollment ended after 229 eligible patients had joined the survey. The STROBE guidelines were consulted when the study was reported.

### 2.3 Data collection

Questionnaires were administered by two investigators. Both investigators received training before the survey, including explanations of the scales and the wording to be used when communicating with participants. The purpose of the study and the method for completing the questionnaire were explained before distribution. Participants filled in the forms independently in a quiet setting. If a participant did not understand an item, the investigator explained its literal meaning without suggesting an answer. Each questionnaire was checked when returned so that unanswered items could be identified. The same procedure was used throughout data collection. Participation could be discontinued at any time, and identifying information was not included in the dataset.

### 2.4 Instrument

#### 2.4.1 General demographic information questionnaire

A researcher-designed form was used to record participant characteristics identified from the relevant literature. The demographic information comprised age, education, occupation, marital status, medical insurance, monthly household income, and residence. Information concerning surgery, cancer stage, chemotherapy, endocrine therapy, targeted therapy, and time since diagnosis was also collected.

#### 2.4.2 The shortened Chinese version of the Family Resilience Assessment Scale (FRAS-C)

Family resilience was quantified with the Shortened Chinese Version of the Family Resilience Assessment Scale (FRAS-C), which was translated and revised by Yuli et al. (2016). Its 32 items are allocated to Holding Positive Perceptions (6 items), Family Communication and Problem-Solving (23 items), and Social Resource Utilization (3 items). Responses receive 1, 2, 3, or 4 points, corresponding to levels from strongly disagree to strongly agree. Summing the items produces a score between 32 and 128, with a larger value denoting stronger family resilience. The reported Cronbach's α for the instrument was 0.96. For the current sample, Cronbach's α was 0.960.

#### 2.4.3 Body Image Scale (BIS)

Body image was measured with the Body Image Scale (BIS) developed by Hopwood et al. (2001). The instrument contains 10 questions addressing emotional, cognitive, and behavioral responses to treatment- or surgery-related changes in appearance. Response options extend from 0, indicating “completely not bothered,” to 3, indicating “extremely bothered.” Possible summed scores range from 0 to 30. Increasing scores reflect increasing body image dissatisfaction. Cronbach's α was reported as 0.90 for the original instrument and was 0.903 in the present sample.

#### 2.4.4 Psychosocial adaptation questionnaire

Psychosocial adaptation was evaluated using the Psychosocial Adaptation Questionnaire developed by Cheng Ran (Cheng, 2010). The questionnaire contains 44 questions distributed across self-esteem and self-acceptance, anxiety/depression, attitude, sense of belonging, and self-efficacy. Responses are assigned 1–5 points, giving a possible sum of 44–220. Total scores are interpreted as low (44–132), moderate (133–175), or high (176–220) psychosocial adaptation. Cronbach's α was 0.902 in the original report and 0.950 for the present dataset.

### 2.5 Statistical analysis

The analysis combined descriptive statistics, group comparisons, correlation testing, and structural equation modeling (SEM). SPSS 25.0 was used for the descriptive and inferential analyses, while AMOS 28.0 was used for model estimation. Distributional characteristics of continuous measures were checked through skewness, kurtosis, and Q–Q plots. Variables satisfying the normality criteria were summarized by the mean and standard deviation. Counts and percentages were used for categorical data. Differences in psychosocial adaptation between two-category variables were examined by independent-samples *t*-tests; variables containing more than two categories were examined by one-way analysis of variance. Pearson's *r* was calculated for pairwise relationships among family resilience, body image, and psychosocial adaptation.

The hypothesized indirect pathway was tested by specifying family resilience as the predictor, body image as the mediator, and psychosocial adaptation as the outcome (Figure 1). Confirmatory factor analysis preceded evaluation of the structural paths. Maximum likelihood estimation was applied, and full information maximum likelihood (FIML) was specified for missing observations. The indirect effect was evaluated from 5,000 bootstrap samples. Evidence of mediation was defined as a bias-corrected 95% confidence interval that remained entirely above or below zero.

**Figure 1:**
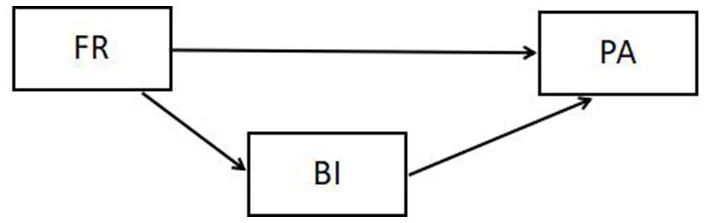
The hypothesized model of family resilience (FR), body image (BI), and psychosocial adaptation (PA) in breast cancer patients.

Overall model adequacy was judged from χ^2^/df together with the root mean square error of approximation (RMSEA), goodness-of-fit index (GFI), adjusted goodness-of-fit index (AGFI), comparative fit index (CFI), Tucker–Lewis index (TLI), normed fit index (NFI), and incremental fit index (IFI). Statistical tests were two-sided, with *P* < 0.05 used as the significance threshold.

## 3 Results

### 3.1 Sociodemographic and clinical characteristics of the participants

Of the 229 patients initially enrolled, two were excluded because of incomplete data and seven because of invalid questionnaires. The final analysis therefore included 220 participants, corresponding to a valid questionnaire response rate of 96.07%. The mean participant age was 43.52 ± 7.30 years, and 123 participants (55.9%) were younger than 45 years. Six participants (2.7%) were unmarried, 205 (93.2%) were married, and nine (4.1%) were divorced or widowed. A total of 83 participants (37.7%) resided in rural areas, whereas 137 (62.3%) resided in urban areas. The mean time since diagnosis was 6.03 ± 2.80 months. Further sociodemographic and clinical characteristics are presented in Table 1.

**Table 1:** Univariate analysis of psychosocial adaptation scores according to participant characteristics ± (*N* = 220).

Variables	Categories	*N*	psychosocial adaptation, mean ±SD	*t/F*	*P*
Age(year)	< 40	71	177.06 ± 29.27	1.486	0.139
	40–59	149	183.03 ± 27.22		
Educational level	Primary school or below	6	179.86 ± 28.74	0.142	0.935
	Junior high school	74	180.34 ± 26.58		
	High school or technical secondary school	44	182.89 ± 30.99		
	College degree or above	96	181.10 ± 27.97		
Type of Medical Insurance	Employee medical insurance	109	183.01 ± 25.52	1.001	0.318
	Resident medical insurance	111	179.23 ± 30.17		
Marital status	Have spouse	205	182.71 ± 27.20	3.219	0.001
	Have no spouse	15	159.13 ± 29.99		
Occupation	Unemployed	54	174.69 ± 31.21	1.534	0.207
	Farmer	42	181.09 ± 26.53		
	Office staff	103	183.12 ± 27.95		
	Retired	21	187.76 ± 19.41		
Primary place of residence	Rural area	84	178.76 ± 31.17	0.968	0.334
	Urban area	136	182.53 ± 25.85		
Average monthly household income (yuan)	≤ 3,000	66	175.30 ± 30.07	2.256	0.083
	3,001–5,000	77	182.44 ± 28.24		
	5,001–8,000	61	182.24 ± 25.73		
	>8,000	16	194.25 ± 21.57		
Surgical type	Modified radical mastectomy	75	179.36 ± 27.95	1.799	0.168
	Breast reconstruction	40	175.35 ± 30.14		
	Breast-conserving surgery	105	184.54 ± 26.89		
Cancer stage	0	9	185.55 ± 27.63	0.809	0.206
	I	48	185.46 ± 25.04		
	II	124	180.93 ± 27.51		
	III	35	175.88 ± 32.46		
	IV	4	170.00 ± 27.79		
Chemotherapy	No	28	187.36 ± 27.79	1.268	0.206
	Yes	192	180.19 ± 27.95		
Targeted Therapy	No	157	180.83 ± 27.29	0.226	0.822
	Yes	63	181.78 ± 29.79		
Endocrine Therapy	No	85	180.93 ± 27.13	0.074	0.941
	Yes	135	181.21 ± 28.58		
Duration Since Diagnosis (months)	≤ 3	34	189.03 ± 23.81	1.811	0.146
	4–6	95	180.02 ± 27.08		
	7–12	83	177.95 ± 30.03		
	>12	8	193.00 ± 27.97		

### 3.2 Scores for family resilience, body image, and psychosocial adaptation

Evaluation of skewness and kurtosis values and inspection of the Q–Q plots indicated that the scores for family resilience, body image, and psychosocial adaptation were normally distributed. The mean scores for family resilience, body image, and psychosocial adaptation were 102.34 ± 12.93, 7.19 ± 5.91, and 181.10 ± 27.97, respectively. Descriptive statistics for the scales and their dimensions are provided in Table 2.

**Table 2:** Descriptive statistics for family resilience, body image, and psychosocial adaptation (*N* = 220).

Variables	Items	Score range	mean ±SD
Family resilience	32	32~128	102.34 ± 12.93
Family communication and problem-solving	23	23~92	73.83 ± 9.39
Social resource utilization	3	3~12	8.57 ± 1.99
Holding positive perceptions	6	6~24	19.94 ± 2.68
Body image	10	0~30	7.19 ± 5.91
Emotional	4	0~12	3.29 ± 2.39
Cognitive	4	0~12	2.73 ± 2.68
Behavioral	2	0~6	1.17 ± 1.39
Psychosocial adaptation	44	44~220	181.10 ± 27.97
Anxiety/depression	8	8~40	30.64 ± 6.88
Self-esteem and self-acceptance	10	10~50	41.30 ± 7.68
Attitude	8	8~40	33.64 ± 5.42
Self-efficacy	9	9~45	38.22 ± 5.26
Sense of belonging	9	9~45	37.30 ± 5.88

### 3.3 Correlations among family resilience, body image, and psychosocial adaptation

Pearson correlation analysis showed that family resilience was inversely correlated with body image (*r* = −0.292, *P* < 0.01) and positively correlated with psychosocial adaptation (*r* = 0.480, *P* < 0.01). Body image was also inversely correlated with psychosocial adaptation (*r* = −0.517, P < 0.01). The correlation matrix is presented in Table 3.

**Table 3:** Pearson's correlations among family resilience,body image and psychosocial adaptation scores (*N* = 220).

Variables	Family resilience	Body image	psychosocial adaptation
Family resilience	1		
Body image	−0.292^**^	1	
Psychosocial adaptation	0.480^**^	−0.517^**^	1

^**^P < 0.01

### 3.4 Measurement model

The measurement model comprised three latent variables represented by 11 observed variables. The factor loadings for the observed variables are presented in Table 4. Confirmatory factor analysis showed that the average variance extracted (AVE) for each construct exceeded 0.50 and that all construct reliability (CR) values exceeded 0.70 (Afthanorhan et al., 2020; Wu, 2010), supporting the convergent validity of the measurement model.

**Table 4:** Results of the confirmatory factor analysis.

Latent variable	Item	Factor load (FL)	Average variance extracted (AVE)	Construct reliability (CR)	Discriminmant validity
Family resilience	FR1	1.013	0.675	0.855	0.822
	FR2	0.561			
	FR3	0.828			
Body image	BI1	0.852	0.733	0.897	0.856
	BI2	0.934			
	BI3	0.776			
Psychosocial adaptation	PA1	0.835	0.763	0.792	0.873
	PA2	0.923			
	PA3	0.897			
	PA4	0.912			
	PA5	0.792			

### 3.5 Structural model and mediation analysis

Based on the measurement-model results, an SEM was constructed in AMOS version 28.0 to test the proposed relationships. Family resilience was specified as the predictor, body image as the mediator, and psychosocial adaptation as the outcome. To preserve model parsimony and focus on the hypothesized relationships among the psychological constructs, demographic and clinical variables were not included as covariates in the final model. The standardized path coefficients are displayed in Figure 2, and the model-fit indices are presented in Table 5. All indices met the recommended criteria, indicating an acceptable fit to the observed data (χ^2^/df = 1.843, CFI = 0.982, and RMSEA = 0.062). Family resilience was inversely associated with body image (β = −0.320, *P* < 0.001) and positively associated with psychosocial adaptation (β = 0.315, P < 0.001). Body image was inversely associated with psychosocial adaptation (β = −0.472, *P* < 0.001). The complete path estimates are reported in Table 6. Modification indices were examined, but no *post hoc* modifications were applied.

**Figure 2:**
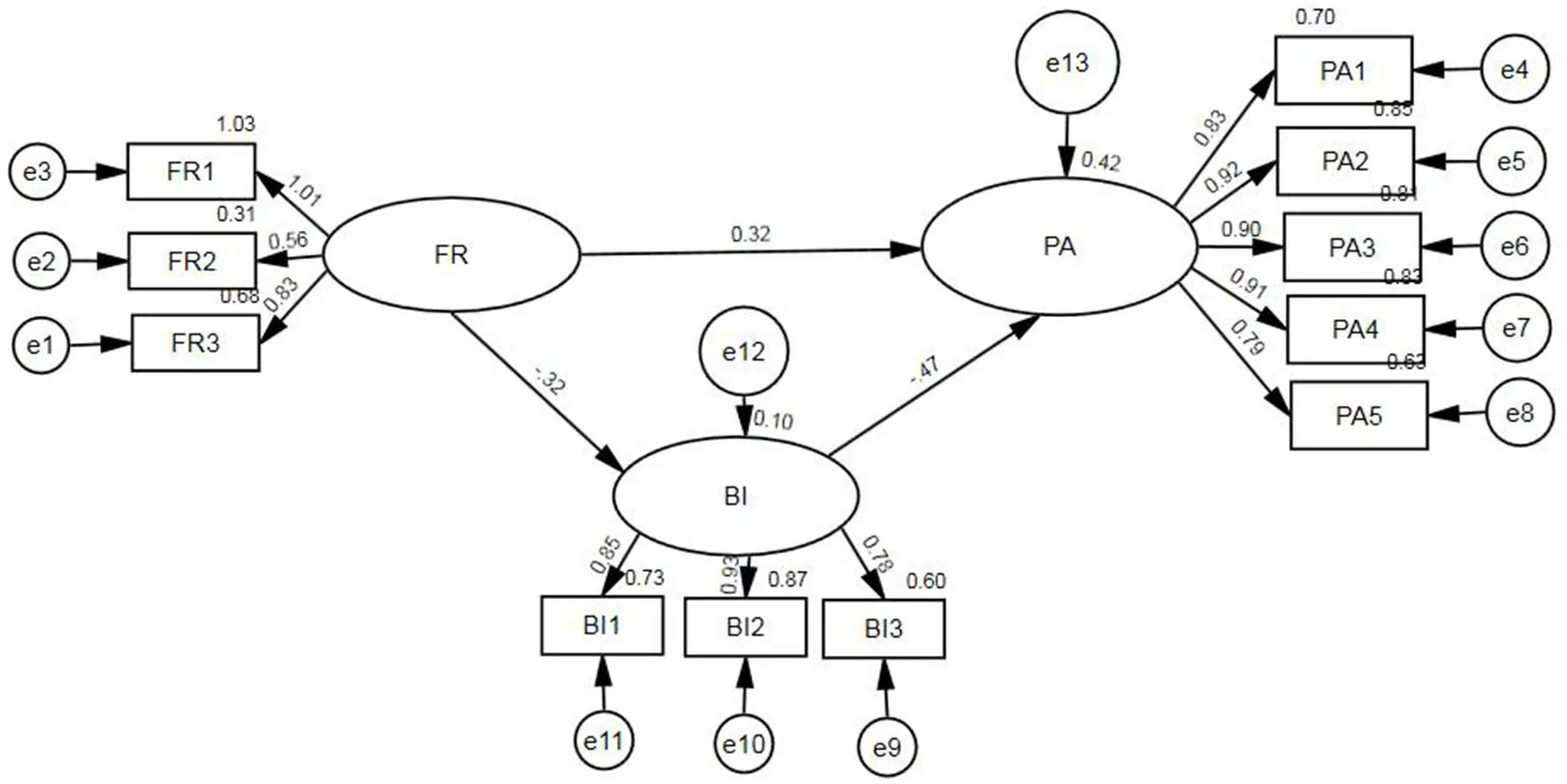
Structural equation model of the relationships among family resilience, body image, and psychosocial adaptation. FR, family resilience; FR1, Family Communication and Problem-Solving; FR2, Social Resource Utilization; FR3, Holding Positive Perceptions; BI, body image; BI1, Emotional; BI2, Cognitive; BI3, Behavioral; PA, psychosocial adaptation; PA1, Anxiety/Depression; PA2, Self-esteem and Self-acceptance; PA3, Attitude; PA4, Self-efficacy; PA5, Sense of belonging.

**Table 5:** Model-fit indices for the structural equation model.

Index	χ^2^/df	RMSEA	GFI	NFI	IFI	TLI	CFI
Result	1.843	0.062	0.942	0.961	0.982	0.975	0.982
Standard value	< 3.000	< 0.080	>0.900	>0.900	>0.900	>0.900	>0.900

χ^2^/df, chi-square/degrees-of-freedom ratio; RMSEA, root mean square error of approximation; GFI, goodness-of-fit index; NFI, normed fit index; IFI, incremental fit index; TLI, Tucker–Lewis index; CFI, comparative fit index.

**Table 6:** Path estimates for the structural equation model.

Path		Path coefficient (non-standardization)	Estimates (standardization)	SE	C.R	*P*
Body image	Family resilience	−0.156	−0.320	0.034	−4.557	< 0.001
Psychosocial adaptation	Body image	−2.501	−0.472	0.366	−6.838	< 0.001
Psychosocial adaptation	Family resilience	0.815	0.315	0.157	5.2	< 0.001

C.R, critical ratios; SE, standard error.

The mediating role of body image was evaluated using 5,000 bootstrap resamples. The 95% CIs for the direct, indirect, and total effects did not include zero, indicating that all three effects were statistically significant. The total effect of family resilience on psychosocial adaptation was 1.206, consisting of a direct effect of 0.815 and an indirect effect of 0.391 through body image. The indirect pathway accounted for 32.42% of the total effect, indicating partial mediation (Table 7).

**Table 7:** Analysis of the mediating effect of body image between family resilience and psychosocial adaptation scores.

Effect	Effect size	*P*	95% CI	Relative effect ratio (%)
Indirect effect	0.391	0.001	0.212~0.643	32.42
Direct effect	0.815	0.002	0.503~1.121	67.58
Total effect	1.206	0.002	0.885~1.515	-

## 4 Discussion

This study examined the associations among family resilience, body image, and psychosocial adaptation in young and middle-aged Chinese patients with breast cancer following surgery. The findings showed significant associations among all three variables. In addition, body image partially mediated the association between family resilience and psychosocial adaptation, supporting the proposed theoretical framework.

The mean psychosocial adaptation score was 181.10 ± 27.97, which was higher than that reported by Chen et al. (2026). One possible explanation is that the participants were in the postoperative recovery period, with a mean time since diagnosis of approximately 6 months. Zhang et al. (2024) followed patients with breast cancer longitudinally and observed an improvement in psychosocial adaptation between 3 and 6 months after surgery. Among the dimensions of psychosocial adaptation, anxiety/depression had the lowest score, suggesting that emotional difficulties remained present during postoperative recovery. This finding is broadly consistent with the observation by Zhang et al. (2026) that 22.5% of patients remained on a trajectory of severe maladaptation. Clinical staff should therefore assess psychosocial adaptation at different stages of recovery and provide appropriate intervention when required. Participants with a spouse had higher psychosocial adaptation scores than those without a spouse. Patients with a spouse may receive greater emotional and practical support, further indicating the importance of the family environment in psychosocial adaptation. However, because relatively few participants were included in the group without a spouse, this finding should be verified in larger samples.

The mean family resilience score was 102.34 ± 12.93, indicating a relatively high level and agreeing with the findings of Zhuo et al. (2025). This result suggests that most participating families had a substantial capacity to cope with adversity. Nevertheless, the Social Resource Utilization dimension had a comparatively low score. Although these families may have maintained internal cohesion, they appeared less proactive in seeking external resources and social assistance. As an immediate source of support, the family may help patients manage emotional and physical difficulties. This interpretation is consistent with previous evidence linking greater family resilience to fewer physical symptoms among patients with cancer (Fu et al., 2024).

The SEM results showed that family resilience was directly associated with psychosocial adaptation and indirectly associated with it through body image. The direct and indirect effect estimates were 0.815 and 0.391, respectively. These findings provide evidence of a possible pathway linking the family environment with psychosocial adaptation through patients' perceptions of their bodies. Previous research has suggested that family resilience may support postoperative recovery by improving patients' adherence to physical activity (Chang et al., 2022). Consistent with this evidence, the present results underline the importance of the family as part of the recovery environment for patients with breast cancer.

Body image partially mediated the association between family resilience and psychosocial adaptation. The mean body image score was 7.19 ± 5.91. Greater family resilience was associated with less body image distress, possibly because resilient families provide more effective communication and emotional support, which may help patients cope with negative perceptions of treatment-related changes in appearance (Hou et al., 2024). Greater body image distress was, in turn, associated with poorer psychosocial adaptation. Family support may therefore be linked to reduced anxiety concerning body image and greater confidence in resuming family and social activities. This finding is consistent with previous research identifying body image as a predictor of psychosocial adaptation among patients with breast cancer (Yoon and Ryu, 2025). However, because the present study was cross-sectional, the temporal order and causal direction of these relationships cannot be established.

### 4.1 Limitations

Participants came from one hospital and were not selected randomly, so they may not represent patients treated in other settings. In addition, all measurements were collected at one time point. The direction of the observed relationships therefore remains uncertain. Responses were provided by the patients themselves and may have been affected by socially desirable answering or by the use of the same measurement method. The SEM did not include surgical type, marital status, chemotherapy status, or other possible covariates. This is relevant because marital status was related to psychosocial adaptation in the univariate analysis, although only a small number of participants did not have a spouse. A larger study would be needed to examine that subgroup separately. The participants were also all Chinese. Family relationships and responses to illness are influenced by culture, and the present results may not apply in the same way to patients from other cultural backgrounds.

## 5 Conclusion

Family resilience was positively associated with psychosocial adaptation, and body image partially mediated this association. Healthcare professionals should consider both the family environment and patients' body image when assessing psychosocial adaptation. Interventions designed to strengthen family resilience and alleviate body image distress may help improve psychosocial adaptation among young and middle-aged patients with breast cancer.

## Data Availability

The original contributions presented in the study are included in the article/supplementary material, further inquiries can be directed to the corresponding author.
